# Mental health of asylum seekers: a cross-sectional study of psychiatric disorders

**DOI:** 10.1186/1471-244X-12-114

**Published:** 2012-08-17

**Authors:** Martina Heeren, Julia Mueller, Ulrike Ehlert, Ulrich Schnyder, Nadia Copiery, Thomas Maier

**Affiliations:** 1 Department of Psychiatry and Psychotherapy, University Hospital Zurich, Culmannstrasse 8, 8091, Zurich, Switzerland; 2 Institute of Psychology, Clinical Psychology and Psychotherapy, University of Zurich, Binzmuehlestrasse 14/26, 8050, Zurich, Switzerland; 3 Psychiatric Services of the Canton St. Gallen-North, Wil, Switzerland

## Abstract

**Background:**

Asylum procedures are known to be protracted, stretching to over ten years in many host countries. International research shows high levels of distress for asylum seekers. Little is known about actual psychiatric morbidity in this population, especially during the first few years postmigration.

**Methods:**

The mental health status of two groups of asylum seekers was assessed: Group 1 (n = 43) had arrived in Switzerland 2.9 (SD 1.1) months prior to assessment, while Group 2 (n = 43) had arrived 15.5 (SD 3.2) months prior to assessment. Psychiatric disorders were diagnosed using the Mini International Neuropsychiatric Interview (MINI). Symptom severity of posttraumatic stress disorder (Posttraumatic Diagnostic Scale), anxiety (Hopkins Symptom Checklist), depression (Hopkins Symptom Checklist), and pain (Verbal Rating Scale) were assessed using self-report questionnaires. Postmigratory factors such as German language proficiency and social contacts were also assessed. Interviews were conducted with the assistance of trained interpreters.

**Results:**

Four out of ten participants met diagnostic criteria for at least one DSM-IV disorder. Groups did not differ with respect to psychiatric morbidity or symptom levels. Major depression (31.4%) and PTSD (23.3%) were diagnosed most frequently. The number of experienced traumatic event types was highly correlated with psychiatric morbidity.

**Conclusions:**

Psychiatric morbidity in asylum seekers in the first two years after arrival is high, with no indication of a decrease in mental distress over time. Traumatic experiences seem to play a major role in morbidity during this time. Considering the magnitude of clinically relevant distress, a short psychological screening upon arrival with a focus on traumatic experiences may be warranted.

## Background

Heightened international research in the area of refugee mental health has highlighted the particular vulnerability of asylum seekers. Distress and psychiatric morbidity are known to be high in this population, with posttraumatic stress disorder (PTSD) rates ranging from 20% to 40% and anxiety and depression rates from 30% to 70% having been reported [1-3].

In most countries, asylum seekers are not screened for mental health problems at any point during the asylum procedure. Consequently, it is not surprising that recent findings on Iraqi asylum seekers in the Netherlands indicate that this population receives very little specific psychiatric treatment [4].

Earlier refugee studies found that mental health improved after the first emotionally turbulent time following resettlement, with postmigration factors playing a relatively small role compared to premigration trauma [5]. However, compared to refugees a decade ago, asylum seekers today are confronted with more unfavourable conditions, one of these being prolonged temporary residency with an uncertain outcome of the asylum procedure [6].

Two studies have focused on the issue of prolonged status insecurity. The first [7] compared two groups of Iraqi asylum seekers who had been in the asylum procedure for either less than six months or more than two years. The authors found a higher prevalence of psychiatric disorders in asylum seekers who had arrived longer ago than in the recently arrived, with significantly higher prevalence rates of anxiety, depressive and somatoform disorders in the former group. All types of psychopathology were predicted by group membership. A recent Australian study compared mental health and living difficulties of asylum seekers holding temporary visas with those having received permanent visas [8]. The authors found an increase in mental health problems and social isolation in temporary holders over a two-year period, whereas permanent holders reported an improvement in mental health.

Based on these findings of increasing mental health problems over a longer period of time spent in uncertainty, we wished to gain a better understanding of psychiatric morbidity during a more restricted period after arrival in the host country. A Swedish longitudinal study on mass-evacuated subjects from Kosovo [9] found that eighteen months after evacuation, PTSD rates had risen from 37% to 80% in subjects not yet having been repatriated, whereas those having repatriated voluntarily showed less morbidity at 52%. Assessing a similar time span, we wanted to address the question of psychiatric morbidity in asylum seekers within the first two years in the host country, and to place psychiatric morbidity in relation to pre- and postmigratory living circumstances. We expected lower levels of morbidity in our samples as compared to mass-evacuated victims of civil war, but a similar difference in symptoms between asylum seekers with a shorter and longer duration of stay in Switzerland.

## Methods

### Participants and procedure

This study was approved by the ethics committee of the canton of Zurich. As part of a pilot study for a planned larger-scaled assessment on mental health and healthcare utilization in asylum seekers [10], two groups of subjects residing in the canton of Zurich were drawn from the national register of adult asylum seekers in Switzerland, based on their length of stay in the country. Group 1 comprised asylum seekers (n = 43) who had arrived in Switzerland 0 to 5 months prior to assessment (mean = 2.9 months, SD = 1.1), and group 2 comprised asylum seekers (n = 43) who had arrived 12 to 26 months prior to assessment (mean = 15.5 months, SD = 3.2). Both groups were sampled consecutively by the Swiss Federal Office for Migration. For each sample, lists of eligible participants were provided every two weeks for six months.

All individuals eligible for inclusion in Group 1 were housed in asylum centres. These centres offer government funded temporary accommodation from which asylum seekers are free to move around in the community. They were approached by centre staff and asked to participate, with their anonymity being guaranteed. Of the 59 eligible subjects approached, 43 (72.8%) gave their written informed consent to participation. Reasons for refusal to volunteer were lack of time (n = 7) and indifference (n = 4), while five subjects did not state a reason. All subjects who agreed to participate completed the assessment and were included in the analysis. All Group 1 assessments were conducted at the asylum centres between August 2008 and January 2009. All subjects’ asylum claims were undergoing processing at the time of assessment.

All individuals eligible for inclusion in Group 2 had been transferred from asylum centres, which are managed supra-regionally, to the municipalities where they were housed in their own or in shared apartments. They were contacted by letter written in their mother tongue or by telephone via interpreters. Of the 67 individuals approached, 43 (64.2%) agreed to participate. Reasons for refusal to participate in this group were lack of time (n = 15), indifference (n = 5) and distrust (n = 1), while three subjects did not state a reason for refusal. All subjects who agreed to participate completed the assessment and were included in the analysis. All of their asylum claims were still being processed at the time of assessment. Group 2 assessments were conducted at the Department of Psychiatry and Psychotherapy, University Hospital Zurich, or at the participants’ homes from November 2008 to April 2009.

All participants gave written informed consent to participate in the study. Interviews were conducted in the participants’ native language with the assistance of trained interpreters. The standardized questionnaires used for the assessment were translated into 10 of the languages most frequently spoken by asylum seekers in Switzerland using established translation procedures [11]. Three subjects did not speak any of the provided languages (Spanish (1), Tibetan (2)). For these subjects, interpreters translated the questionnaire items into their native languages during the interview.

### Instruments

The demographic variables assessed included age, gender, marital status, level of education and region of origin. Premigratory traumatic experiences were assessed using the first sections of the Harvard Trauma Questionnaire [HTQ; [12] and the Posttraumatic Diagnostic Scale [PDS; [13]. Both instruments are widely used in research with refugees [f.e. [14,15]. We used both trauma lists in order to assess the widest possible range of traumatic experiences. To avoid repetition, we removed overlapping items from the HTQ, yielding a final list of 23 items. Only traumatic events experienced or witnessed by the respondents themselves were included in the present study; events which the respondents had only heard about were excluded from the analysis.

The postmigratory factors assessed included length of residence in Switzerland, family status (whereabouts of family members), social contacts, German language proficiency, homesickness, and feeling at home in Switzerland.

Psychiatric disorders (DSM-IV) were assessed using the Mini International Neuropsychiatric Interview [MINI; [16]; Sections A, C to O, R, U]. This instrument has been shown to provide reliable DSM-IV-TR [17] diagnoses within a short time frame and was originally designed for use in epidemiological studies [18]. In this study, we used cluster diagnoses: anxiety disorders comprised panic disorder, agoraphobia, generalized anxiety disorder and social phobia. PTSD was considered separately from other anxiety disorders.

Symptom severity was assessed by means of self-report questionnaires. Section III of the PDS [13] was used to measure symptom severity of PTSD. The 17 items in this section assess symptoms experienced in the month prior to assessment. Items are rated on a 4-point scale, and sum scores range from 0 to 51. The PDS has demonstrated good psychometric properties and is recommended as a useful tool for assessing PTSD symptoms [19].

The Hopkins Symptom Checklist-25 [HSCL-25; [20] was used to measure symptoms of anxiety and depression. This checklist was developed for use in refugee populations. It comprises 10 anxiety items and 15 depression items scored on a 4-point severity scale. Mean scores range from 0 to 4. The instrument has been shown to have good psychometric properties.

The Verbal Rating Scale [VRS, from SF-36; [21] was used to measure pain intensity. Physical pain in the previous month is assessed by a single item, with scores ranging from 0 (‘no pain at all’) to 5 (‘severe pain’). The instrument has been shown to have good reliability and validity [22].

### Data analysis

Data were coded and analyzed using SPSS 19.0. *χ*^2^ tests (for frequencies), and Student’s t*-*tests (for mean scores) were used to compare the two groups on sociodemographic characteristics.

Descriptive statistics were used to analyze demographic data, characteristics of respondents’ living situation in Switzerland and mental health data. Kolmogorov-Smirnov tests were used to analyze whether the interval data were normally distributed. Paired samples t-tests (for normally distributed continuous data), Wilcoxon tests for paired samples (for ordinal data), McNemar-Bowker tests (for nominal data) and McNemar’s tests (for binominal data) were conducted to identify differences between groups. Chi-square and Spearman coefficients were calculated for correlations between mental health outcomes and pre- and postmigratory factors.

## Results

### Demographic characteristics

Both groups comprised more males than females (Table 1). Participants’ ages ranged from 18 to 63 years, with Group 2 participants being significantly older than Group 1 participants (t = -3.4, df = 78.1, p < .01). About half of the participants were married, and 71% of those had come to Switzerland with their spouses. Length of education ranged from 0 to 23 years. Participants came from a wide range of Asian and African countries. All European participants originated from the former Yugoslavia. Whereas almost one third of Group 1 participants originated from Africa and one quarter from Europe, the large majority of Group 2 participants (74.4%) came from Asian countries (*χ*^2^ = 8.3, df = 2, p < .05). Group 1 participants originated from Afghanistan (n = 2), Algeria (n = 1), Cote d’Ivoire (n = 2), Eritrea (n = 2), Guinea (n = 1), Iraq (n = 5), Iran (n = 5), Kosovo (n = 9), Lebanon (n = 1), Liberia (n = 1), Morocco (n = 1), Nigeria (n = 1), Serbia (n = 2), Somalia (n = 1), Sri Lanka (n = 2), Sudan (n = 2), Syria (n = 4), and Tunisia (n = 1). Reported native languages were Albanian (n = 3), Arabic (n = 15), Bambara (n = 1), Bosnian (n = 1), English (n = 1), Farsi (n = 7), French (n = 2), Kru (n = 1), Kurdish (n = 1), Serbian (n = 6), Somali (n = 1), Tamil (n = 2), and Tigrinya (n = 1). Group 2 participants originated from Afghanistan (n = 5), Angola (n = 1), Burkina Faso (n = 1), Cote d’Ivoire (n = 1), Eritrea (n = 2), Iraq (n = 8), Iran (n = 3), Kosovo (n = 2), The Maldives (n = 3), Nigeria (n = 1), Palestine (n = 2), Serbia (n = 4), Sri Lanka (n = 2), Tibet (n = 2), and Turkey (n = 6). Native languages in this group were Albanian (n = 3), Arabic (n = 10), English (n = 2), Farsi (n = 6), Farsi (n = 2), French (n = 3), Igbo (n = 1), Kurdish (n = 1), Portuguese (n = 1), Serbian (n = 3), Tamil (n = 2), Tibetan (n = 2), Tigrinya (n = 2) and Turkish (n = 5). The vast majority (n = 70) reported war/political persecution as reason for migration, three subjects named poverty and four named family reunion as a reason.

**Table 1:** Demographic characteristics and postmigratory variables

**Characteristic**	**Asylum seekers**	
	**Group 1 (n = 43)**	**Group 2 (n = 43)**
Male gender, n (%)	31 (72.1)	29 (67.4)
Age in years, mean (SD)	26.7 (7.2)	32.9 (9.6)
Married, n (%)	17 (39.5)	21 (48.8)
Education in years, mean (SD)	10.2 (4.5)	9.5 (4.9)
Region of origin, n (%)		
Asia	19 (44.2)	32 (74.4)
Europe	11 (25.6)	6 (14.0)
Africa	13 (30.2)	5 (11.6)
Time in Switzerland in months, mean (SD)	2.9 (1.1)	15.5 (3.2)
Family members in Switzerland, n (%)	17 (39.5)	28 (65.1)
Social contacts beyond family, n (%)		
Never	10 (23.3)	8 (15.6)
Sometimes	20 (46.5)	22 (51.2)
Frequently	13 (30.2)	13 (30.2)
German proficiency, n (%)		
Poor	29 (67.4)	13 (30.2)
Medium	13 (30.2)	22 (52.2)
Sufficient	1 (2.3)	8 (18.6)
Homesickness, n (%)		
Never	2 (4.7)	4 (9.5)
Sometimes	18 (44.2)	20 (47.7)
Frequently	22(51.2)	18 (41.9)
Feeling at home in Switzerland, n (%)		
Not at all	14 (32.5)	8 (18.7)
A little	12 (27.9)	14 (32.6)
Quite/very much	17 (39.5)	21 (48.8)

The two groups did not differ significantly in any other sociodemographic characteristics.

### Postmigratory characteristics

At the time of assessment, all study participants were unemployed and dependent on social welfare benefits. 39% of Group 1 and 65% of Group 2 had relatives living in Switzerland (*χ*^2^ = 5.6, df = 1, p < .05). The majority reported having little or no social contact beyond their own family members. Although they had been in the country for longer, Group 2 participants did not report having more social contacts beyond the family than Group 1 (*χ*^2^ = 0.3, df = 2, p = .853). However, they did report a higher level of German proficiency (*χ*^2^ = 13.83, exact p < .01), with 18.6% of Group 2 members rating their German as sufficient for daily use. Although most participants in both groups experienced homesickness (87.1%), almost half (44.2%) reported feeling quite or very much at home in Switzerland. There were no statistically significant differences between the groups in these respects.

### Traumatic events

Premigratory exposure to traumatic events was high in both groups, with an average of almost six different types of traumatic events having been experienced over the lifetime (mean = 5.73, SD = 4.18). Samples did not differ either in presence or frequency of potentially traumatic events experienced. Across the whole sample, 88.4% of participants reported experiencing at least one traumatic event, and the number of event types experienced ranged from 0 to 17. Reported event types are listed in Table 2.

**Table 2:** Lifetime exposure to traumatic events: number and types experienced

**Variables**^**a**^	**Asylum seekers**	
	**Group 1 (0–5 months, n = 43)**	**Group 2 (12–26 months, n = 43)**
Number of traumatic event types experienced, mean (SD)	5.2 (3.9)	6.3 (4.4)
Experienced at least 1 traumatic event, n (%)	39 (90.7)	37 (86.0)
Type of traumatic events experienced, n (%)		
Forced separation from family member	18 (41.9)	19 (44.2)
Unnatural death of family member or friend	17 (39.5)	18 (41.9)
Non-sexual assault by stranger	17 (39.5)	17 (39.5)
Being close to death	14 (32.6)	20 (46.5)
Lack of food or water	18 (41.9)	15 (34.9)
Lack of shelter	14 (32.6)	15 (34.9)
Murder of family member or friend	17 (39.5)	12 (27.9)
Enforced isolation from others	12 (27.9)	15 (34.9)
Non-sexual assault by family member or familiar person	12 (27.9)	14 (32.6)
Combat situation	9 (20.6)	16 (37.2)
Imprisonment	9 (27.9)	15 (34.9)
Torture	11 (25.6)	11 (25.6)
Ill health without access to medical care	11 (25.6)	11 (25.6)
Serious accident	7 (16.3)	11 (25.6)
Murder of one or more strangers	7 (16.3)	10 (23.8)
Serious physical injury	6 (14.0)	10 (23.3)
Disappearance or kidnapping	4 (9.3)	11 (26.2)
Natural disaster	5 (11.6)	7 (16.3)
Life-threatening illness	2 (4.7)	7 (16.3)
Brainwashing	4 (9.3)	5 (11.6)
Sexual assault by family member or familiar person	3 (7.0)	6 (14.0)
Sexual contact as a minor with person 5+ years older	3 (7.0)	5 (12.2)
Sexual assault by a stranger	3 (7.0)	1 (2.4)

^a^ assessed with the trauma lists of the Posttraumatic Diagnostic Scale [PDS; [13] and the Harvard Trauma Questionnaire [HTQ; [12].

### Mental health

Findings on clinician-rated and self-reported mental health outcomes are presented in Table 3. In both groups, the rate of clinician-rated psychiatric morbidity was high, with 4 in 10 participants meeting the DSM-IV criteria for at least one disorder. No significant group differences emerged.

**Table 3:** Psychiatric outcome measures: clinician-rated diagnoses and self-reported symptom severities

**Psychiatric diagnoses and symptom severity**	**Asylum seekers**	
	**Group 1 (0–5 months, n = 43)**	**Group 2 (12–26 months, n = 43)**
PTSD		
Diagnosed PTSD^a^, n (%)	6 (14.0)	14 (32.6)
Symptom severity^b^, mean (SD)	13.6 (12.1)	16.9 (14.4)
Depression		
Diagnosed Major Depression^a^, n (%)	15 (34.9)	12 (27.9)
Symptom severity^c^, mean (SD)	1.9 (0.8)	2.1 (0.6)
Anxiety		
Diagnosed anxiety disorder^a,d^, n (%)	5 (11.6)	2 (4.7)
Symptom severity^d^, mean (SD)	1.8 (0.7)	2.0 (0.8)
Pain		
Diagnosed Pain Disorder^a^, n (%)	3 (7.0)	7 (16.3)
Pain intensity^e^, mean (SD)	1.6 (1.6)	2.4 (1.7)
Alcohol		
Diagnosed alcohol abuse/dependency	3 (7.0)	1 (2.3)
Number of diagnoses^a^		
0	26 (60.5)	26 (60.5)
1	3 (7.0)	4 (9.3)
2	9 (20.9)	5 (11.6)
3 or more	5 (11.6)	8 (18.6)

^a^assessed using the Mini International Neuropsychiatric Interview [MINI; 16; Sections A, C to O, R, U].

^b^assessed using the Posttraumatic Diagnostic Scale [PDS; [13], Part III sum scores (range 0–51).

^c^assessed using the Hopkins Symptom Checklist-25 [HSCL-25; [20], mean scores on items 1–10 (range 0–4).

^d^comprises panic disorder, agoraphobia, generalised anxiety disorder and social phobia.

^e^assessed using the Verbal Rating Scale (VRS, from SF-36; range 0–5).

Major Depression (MD) was diagnosed most frequently. The two groups did not differ significantly regarding diagnostic status or severity of depressive symptoms.

Regarding PTSD, 32.6% of Group 2 participants were diagnosed with PTSD, compared with 14.0% of Group 1. Thus, PTSD rates in Group 2 were more than twice as high as in Group 1 (*χ*^2^ = 4.17, df = 1, p < .05). However, the two groups did not differ in terms of self-reported posttraumatic symptom severity (t = -1.2, df = 81, p = .254).

The two groups did not differ in the frequency of Pain Disorder diagnoses, but Group 2 participants did report significantly higher pain intensity than did Group 1 participants (t = -2.3, df = 82, p < .05). An additional analysis revealed that participants with a diagnosis of PTSD reported higher pain intensity than those without PTSD (t = -3.95, df = 35.4, p < .001).

Other anxiety disorders diagnosed included panic disorder, agoraphobia, generalized anxiety disorder and social phobia. These disorders were less frequent than MD or PTSD. Four participants (4.7%) were diagnosed with alcohol abuse or dependency. The two groups did not differ significantly in their rates of anxiety disorders or alcohol abuse.

### Factors associated with psychiatric outcomes

Next, we focused on the most frequent disorders, MD and PTSD, as well as on overall psychiatric morbidity, i.e. fulfilling diagnostic criteria for at least one of the assessed disorders. In both samples, neither socio-demographic nor assessed post-migratory variables showed significant correlations with MD, PTSD or overall psychiatric morbidity. Duration of stay in the host country also did not correlate with any mental health outcomes. In both samples, a higher number of trauma event types was associated with PTSD (t = -2.62, df = 41, p = .012 for Sample 1 and t = -3.85, df = 41, p = .000 for Sample 2, resp.) and with overall psychiatric morbidity (t = -3.02, df = 41, p = .004 for Sample 1 and t = -3.14, df = 41, p = .003 for Sample 2, resp.). The number of trauma types was associated with MD in Sample 1 only (t = -3.88, df = 41, p = .000).

## Discussion

This is one of the first studies to not only assess levels of distress in asylum seekers, but also to screen for psychiatric disorders, while at the same time addressing possible influences of length of stay, premigration trauma and postmigration factors. To our knowledge, it is the first study to restrict itself to the first two years of the asylum procedure.

The present results show high overall levels of psychiatric morbidity in our samples of asylum seekers, with Major Depression and PTSD being diagnosed most frequently, followed by Pain Disorder and other anxiety disorders. None of the assessed postmigratory factors correlated with psychiatric morbidity; nor did time since arrival in Switzerland. For both samples, the number of trauma types experienced premigration was highly correlated with PTSD and overall psychiatric morbidity.

Results suggest that traumatic events experienced pre- and perimigration strongly influence mental health after arrival in the host country. The social situation seems to neither buffer nor enhance symptoms in this first postmigration period.

The results on mental health in the current sample of asylum seekers are well in line with those of previous studies reporting severe mental distress and high psychiatric morbidity in asylum seekers [3]. As expected, PTSD rates were lower than in massevacuated victims of war [9]. We found comparably higher PTSD rates with a longer duration of stay, but this difference did not show in other psychiatric disorders.

The negative influence of postmigratory living problems on mental health found in previous studies looking at longer time spans since arrival [7] was not found in this study. Instead, traumatic events showed to be associated with psychiatric morbidity. Trauma has been consistently confirmed as a risk factor for refugee mental health [6], especially in the first period after resettlement.

The main strength of the present study lies in its sampling: we were able to follow the lead of previous studies [7] by ensuring the assessment of a random sample from the national register of asylum seekers without excluding those who are socially isolated or do not utilize support services. Since the register includes all persons who seek asylum in Switzerland, we were certain of considering the total population of asylum seekers when drawing our samples. Second, we used structured clinical interviews to assess psychiatric morbidity. This approach allowed face-to-face screening of the sixteen most common DSM-IV-TR psychiatric disorders. The use of this instrument reduced the risk of overestimation of symptom prevalence, as has been observed with self-report instruments [23]. With the assistance of trained interpreters, we were able to resolve any misunderstandings in the questionnaire items. Finally, the demographics of the sample add to the validity of the study: By including asylum seekers of all origins, we ensured that the results were not affected by regional conflict severity or by culture-specific coping styles.

The main limitation of this study lies in its cross-sectional design, which only allows for limited conclusions to be drawn on the course of mental health over time. Comparability of samples was further limited by differences between samples in terms of sociodemographic and postmigration characteristics. Since the presented assessment was conceived as a pilot study for a future large-scale longitudinal study of asylum seekers’ mental health, participants had to be recruited consecutively and could not be matched by age or region of origin. As shown in the analyses, none of the variables with group differences were associated with mental health outcomes, which led us to assume comparability of samples concerning traumatic stress and psychiatric outcomes.

Further limitations relate to the assessment of the participants’ situation in exile. First, our assessment of postmigratory factors was not exhaustive. It included mostly social factors, leaving out factors such as distress caused by immigration office interviews or the ability to cope with postmigration stress. It is therefore unclear whether a more complete set of characteristics of the postmigratory situation may have been shown to interact with mental health on a significant level. Also, we don’t know whether conditions for asylum seekers are more or less supportive in Switzerland relative to other countries. Therefore, we cannot make any inferences regarding the specific impact of the post-migration stress factor in Switzerland. It is possible that conditions in the country of asylum can make a significant difference to the overall impact of post-migration uncertainties about a person’s refugee status. This issue will require further research, using an international, multi-site approach. Second, previous studies show that there are protective factors such as coping mechanisms or security [e.g. [24]. Due to the extensive screening of psychiatric disorders, we did not have the timely capacity to assess the whole scope of these postmigration factors. We chose to limit ourselves to the assessment of adaption in the host country, knowingly leaving out important items on resources and coping mechanisms. The beneficial effect of employment also could not be examined, because asylum seekers at this stage in the procedure generally do not have a job. Future analyses should include a sample of asylum seekers at a later stage in the procedure, who are more likely to be employed. Third, we did not assess adverse life events after arrival in Switzerland, which a previous study has found to influence mental health [7]. It can be argued that by leaving out traumatic events about which the respondents had only heard, we did not assess these events exhaustively. We decided to include only events experienced or witnessed by the subjects themselves in order to avoid creating a ceiling effect in positive answers, since the mean number of reported event types would have almost doubled under inclusion of events about which the respondents had only heard (mean of events heard about = 4.06, SD = 4.42).

Finally, ethical challenges in the questioning of asylum seekers need to be addressed. Having to be considered as a vulnerable group, asylum seekers may feel intimidated or frightened by the interview situation, which may remind them of interviews with Migration Officers or interrogations in their home country. We tried to minimize such associations by employing only trained psychologists to conduct the interviews and by insuring that participants understood at the beginning of the interview that individual data would be anonymized and would not influence the outcome of their asylum request in any way. Where psychiatric morbidity was diagnosed, we informed participants of treatment possibilities, which are unfortunately scarce for asylum seekers and refugees.

To date, empirical studies examining the influence of the postmigratory situation soon after arrival are scarce, making it impossible to clearly determine the local versus global relevance of the present findings. However, Switzerland’s current asylum policies are comparable to those of most other countries implementing mandatory detention. It therefore seems likely that, in countries with similar asylum policies, mental distress in the months after arrival is likewise closely linked to traumatic events experienced prior to migration.

Future research should examine the course of mental health and postmigration characteristics over a longer period of time. Special attention should be given in this respect to the potential mediating effect of postmigratory difficulties on the predictive power of traumatic events on mental health outcomes – specifically, the possibility that individuals traumatized in their home country are more vulnerable to postmigratory stressors. The present study thus highlights the importance of the thorough, large-scale, and longitudinal examination of how postmigratory characteristics impact the mental health of asylum seekers.

## Conclusions

The results of this study show that psychiatric morbidity in asylum seekers in the first two years of the asylum procedure is high and that the extent of psychiatric morbidity does not differ between those who have very recently arrived and those who have been in the country for one to two years. Results suggest that past traumatic experiences play a major role in the emergence and maintenance of mental health problems in this first period after arrival in the host country.

Our results are in line with previous findings suggesting that the mental wellbeing of asylum seekers warrants special attention, and that interventions should be developed to prevent psychiatric morbidity from consolidating and thus precluding asylum seekers’ cultural and economic integration into the host society. A short psychological screening after arrival might prove helpful here. Moreover, carefully designed longitudinal studies with unselected populations of asylum seekers should be undertaken before political recommendations are issued on government interventions and policy changes.

## Competing interests

The authors declare that they have no competing interests.

## Authors’ contributions

MS made substantial contributions to the conception and design, acquisition of data, analysis and interpretation of data, and drafted the manuscript. JM made substantial contributions to the conception and design and critically revised the manuscript. TM made substantial contributions to the conception and design of the study and critically revised the manuscript. NC made substantial contributions to the study design. UE critically revised the conception of the study and the manuscript. US made substantial contributions to the study conception and critically revised the manuscript. All authors read and approved the final manuscript.

## Pre-publication history

The pre-publication history for this paper can be accessed here:

http://www.biomedcentral.com/1471-244X/12/114/prepub
